# Process-Structure-Function in Association with the Main Bioactive of Black Rice Flour Sieving Fractions

**DOI:** 10.3390/foods8040131

**Published:** 2019-04-18

**Authors:** Carmen Alina Bolea, Leontina Grigore-Gurgu, Iuliana Aprodu, Camelia Vizireanu, Nicoleta Stănciuc

**Affiliations:** Faculty of Food Science and Engineering, Dunărea de Jos University of Galati, Domnească Street 111, 800201 Galati, Romania; carmen.bolea@ugal.ro (C.A.B.); leontina.gurgu@ugal.ro (L.G.-G.); iaprodu@ugal.ro (I.A.); camelia.vizireanu@ugal.ro (C.V.)

**Keywords:** black rice, phytochemicals, proteins, sieving, thermal treatment

## Abstract

The aim of this work was to advance knowledge on the potential use of black rice different sieving fractions for various functional applications, through proximate analysis, thermal degradation kinetics of phytochemical and characterization of the thermal behavior of the main proteins, from the perspectives of their use as a food ingredient. The results indicated that the thermal degradation of phytochemicals followed a first-order reaction kinetics for all the tested fractions. The temperature-dependent degradation was adequately modeled according to the Arrhenius equation. The calculated activation energies (*E_a_*) and *k* values were different among the four studied parameters. The kinetic parameters depended on the grinding and sieving degree, the anthocyanins being the most thermolabile compounds, thus affecting the antioxidant activity. Three protein fractions were identified by electrophoresis with different molecular weight, such as albumin, globulin, and glutelin. The fluorescence spectroscopy experiments revealed the sequential character of the heat-induced conformational changes, different molecular events being suggested, such as folding in the lower temperature range and unfolding at higher temperature. The significance of the study is evidenced by the need to identify and advance the process-structure-function relationships for various biologically active compounds from the perspective of obtaining food or ingredients nutritionally optimized.

## 1. Introduction

Diets rich in grains contribute to good health, especially by reducing the risk of chronic diseases such as cardiovascular disease, type II diabetes, obesity, or cancer [1]. Nowadays, there is an increasing demand on the market for high quality protein ingredients. The exploitation of new plant protein sources such as rice could be considered a good choice in terms of nutritional and hypoallergenic properties compared to other cereals and legume proteins [2]. Rice (*Oryza sativa* L.) is one of the world’s most important cereal crops by providing a staple food source for more than 50% of the world’s population [3]. An important advantage in using rice or rice flour in food composition is the absence of gluten, being recommended as an alternative for people suffering from physiological disorders caused by gluten intolerance [4]. Among rice varieties, black rice is very popular in Asia, especially in China [5], as a good source of minerals, and a phytochemical besides the basic nutrients. Phytochemicals are defined as bioactive plant compounds found in fruits, vegetables, whole grains, and other plant foods and are classified as carotenoids, phenolics, nitrogen-containing compounds, and organosulfur compounds [6]. For example, Zhang et al. [7] stated that the whole grain phytochemicals include carotenoids (lutein, zeaxanthin, β-cryptoxanthin, and β-carotene), phenolics, and vitamin E. Phenolics include phenolic acids (*p*-coumaric, caffeic, ferulic, vanillic, and syringic acids) and flavonoids (flavonols, flavones, catechins, and anthocyanins). Due to the high phytochemical content, the whole grain possesses a significant antioxidant activity being able to scavenge for free radicals that may increase the oxidative stress and potentially damage large biological molecules, such as lipids, proteins, and DNA [8].

Rice is consumed as a staple food as it is or in a milled form [9]. However, when incorporating rice as an ingredient in different food, it is important to use fine flour with a small particle size. For example, the use of natural pigments like anthocyanins as coloring agents in food products is receiving, nowadays, an increasing attention as anthocyanins become more and more attractive to consumers due to significant positive health benefits [10]. 

In the food industry, grinding is a procedure that is part of a large set of operations involved in the downsizing process [11]. The grinding of rice flour may affect the physicochemical and phytochemical content by creating differences in the distribution models of the particle size. Additionally, thermal processing may greatly influence the content of bioactive compounds and proteins, due to their high vulnerability towards different processing factors such as: pH, light, thermal treatment, enzymes, oxygen, and copigments [12]. For instance, thermal treatments often lead to substantial denaturation of the native structure of food proteins, which is critical to protein functionality. 

There are many studies involving thermal degradation of anthocyanins from juices, concentrates, and extracts from fruits, such as blackberry [13], sour cherry extract [14], strawberry [15], sweet cherries, and plum extracts [16,17], black rice extract [18], etc. Because of the inherent use of heat in food processing, the knowledge of heat-induced changes on bioactives, including both phytochemicals and proteins, could provide advanced information on the structure-function relationships that can be correlated to the desired functional and nutritional properties of the final food [19]. However, from the perspectives of using these biologically active compounds as food ingredients, no data could be found regarding the content in various black rice sieving fractions or in terms of their mechanisms and kinetics of thermal degradation.

Therefore, this study was undertaken to assess the chemical and phytochemical composition of the seven fractions of rice bran obtained after sieving, which were further extracted and subjected to a heating process at temperatures ranging from 60 to 100 °C for different preset heating times (0–20 min). Thermal degradation with reference to total polyphenol content (TPC), total flavonoids (TFC), total monomeric anthocyanins (TAC), and antioxidant activity was described using kinetic models. Additionally, several protein fractions were extracted and their thermal behavior was characterized by fluorescence spectroscopy means. The fluorescence spectroscopy technique was used to determine how the thermal treatment, which is commonly used for food processing, affects the proteins’ behavior, and in consequence their functional properties. These data would be very useful in predicting the potential utilization of the different sieving fractions from the black rice flour in as ingredients in specific food products.

## 2. Materials and Methods 

### 2.1. Black Rice Sample 

The black rice (*Oryza sativa L, var. japonica*) was purchased from a local market, Galati (Romania). The black rice was grinded in a laboratory mill (WZ-2, Sadkiewicz, Poland) to obtain the whole meal black rice flour (F), which was then sifted through several sifts with different meshes hence obtaining seven fractions (F1–F7). The sieves diameters were: 630 µm, 550 µm, 315 µm, 180 µm, 125 µm, 90 µm and <90 µm. The refusal from each sieve was further sieved using the subsequent sieve with a lower mesh. Thus, the refusal obtained through the 630 µm sieve was the F1 black rice flour while F2 flour stream had a particle size ranging between 550 and 630 µm, F3 flour stream had a particle size ranging between 315 and 550 µm and so on, in the end F7 had a particle size lower than 90 µm. 

### 2.2. Proximate Chemical Composition

The black rice fractions were characterized in terms of composition using the following methods: moisture content using the AOAC method (1990), proteins through Kjeldahl method (AACC method 46–13), lipids through Soxhlet extraction, ash content with STAS 90/1988 method, and crude fiber using AOAC Official Method 962.09. The carbohydrates content was estimated by subtracting the proteins, ash, lipids and fibers from the dry weight of the samples.

### 2.3. Phytochemicals Extraction

For the extraction of phytochemicals, 1 g of flour or different size fractions were mixed with 70% ethanol (8 mL) and allowed to stand for 24 h. The mixture was centrifuged at 12,000× *g*, at 4 °C for 10 min. The supernatant was collected and concentrated at 40 °C to dryness (Rotavapor R-124, Buchi, Switzerland) and used to estimate the phytochemical content in terms of TAC, TPC, TFC and antioxidant activity. For further experiments, the extracts were dissolved in ultrapure water.

### 2.4. Phytochemicals Analysis

The TPC, TFC, TAC and antioxidant activity, expressed as 2,2-diphenyl-1-picrylhydrazyl radical scavenging activity (DPPH-RSA) of the extracts were determined as described by Bolea et al. [18]. 

### 2.5. Proteins Extraction

5 g of black rice flour with the particle size lower than 90 µm (F7) was suspended in 200 mL of distilled water, and was homogenized for 3 h at 25 °C. The suspension was centrifuged at 3000× *g* for 30 min, and the albumin containing supernatant (further called albumin fraction–ALB) was collected. The globulins fractions (GLO) were then solubilized from the collected residue after the first centrifugation step, using 200 mL of 5% NaCl. The GLO was separated through centrifugation at 3000× *g*, for 30 min. The residue was afterwards treated with 1 M NaOH for 1 h and centrifuged under the same conditions, to obtain the glutelin fraction (GLU). This final residue was mixed with 70% ethanol and mixed for 1 hour, to obtain the prolamin fraction.

### 2.6. Black Rice Proteins Separation through SDS-PAGE 

The separation of the protein fractions from black rice flour with the particle size lower than 90 µm was achieved by sodium dodecyl sulfate–polyacrylamide gel electrophoresis (SDS-PAGE), using a 4.5–15% acrylamide gradient gel. The samples were denatured by heating at 95 °C in the presence of β-mercaptoethanol. The molecular weight marker, Precision Plus Protein Dual Extra (BioRad, Hercules, CA, USA) was used to estimate the molecular weights of the rice proteins extracted in different solvents. The protein bands were stained with Coomassie Brilliant Blue R-250 (Bio-Rad, Hercules, CA, USA).

### 2.7. Heat Treatment

The black rice proteins fractions were solubilized in a Tris-HCl buffer (0.02 M), pH 7.7 and thermally treated for 10 min at temperatures ranging from 25 °C to 100 °C. For the thermal degradation kinetic studies of phytochemical, the tubes were heated in the same temperature range of 60 °C to 100 °C for different treatment times (0–20 min). The thermal treatment experiments were conducted in a thermostatic water bath (Digibath-2 BAD 4, RaypaTrade, Barcelona, Spain). After the thermal treatment, the Eppendorf tubes were immediately cooled in ice bath in order to prevent further degradation. 

### 2.8. Kinetic Data Analysis

The degradation kinetics of phytochemical was described by fitting the experimental data to a first order kinetic model (Equation (1)):

(1) $$\frac{C}{C_{0}}=e^{-kt},$$

where, *C* is the parameter to be estimated, the subscript *0* indicates the initial value of the parameter, *t* is the heating time, and *k* is the rate constant at temperature *T* (1/min). The Arrhenius model was used to describe the temperature dependence of the degradation rate constants as shown by Turturică et al. [16].

### 2.9. Fluorescence Spectroscopy Measurements

The fluorescent properties of the proteins from black rice flour were measured using the LS-55 luminescence spectrometer (PerkinElmer Life Sciences, Shelton, CT, USA). A volume of 500 μL of thermally treated proteins samples was diluted to 3 mL using 10 mM phosphate buffer (pH 7.0).

#### 2.9.1. Intrinsic Fluorescence

The fluorescence spectra were collected for the excitation wavelengths of 274 nm, 280 nm, and 292 nm. The scanning speed was 1000 nm/min and the slits 10 nm.

#### 2.9.2. Quenching Experiments

Fluorescence quenching experiments were performed for all the investigated protein fraction using 5 M potassium iodide (KI) and 8 M acrylamide as quenchers at the excitation wavelength of 292 nm, while the emission was collected in the 310–420 nm range. Aliquots (25–150 µL) of freshly prepared stock solutions of quenchers were added into the cuvettes that contained buffer and protein solution. Data were analyzed through linear regression analysis of the experimental data as previously described Dumitrașcu et al. [20].

#### 2.9.3. Synchronous Spectra

The characteristic features of tyrosine and tryptophan residues were obtained after setting Δ*λ* at 15 nm and 60 nm.

### 2.10. Statistical Analysis

The experiments were performed in triplicates and the results are reported as mean values. Statistical analysis of experimental data was performed using the Microsoft Excel software. The Anova method was employed to assess the differences between the investigated variables and post hoc analysis via Tukey method was further used when *p*-value in Anova analysis was lower than 0.05.

## 3. Results and Discussion

### 3.1. Proximate Analysis of Chemical Composition of The Rice Milled Fractions

The proximate compositions of black rice flour and its different fractions are shown in Table 1, which was calculated on a dry basis to allow comparison with data from the literature. Moisture content was found to be ~11–11.5 % for all fractions. The obtained results are within the acceptable limit (12%) recommended for long term storage, value which allows it to avoid insect infestation and microbial growth [21]. The moisture content of some flavored rice varieties analyzed by Asaduzzaman et al. [22] varied between 11.25% and 15.13%. Saikia et al., [23] reported for different milled aromatic rice samples values varying from 11.6 ± 04% to 13.7 ± 0.12%, whereas Sompong et al. [24] suggested values varying from 9.28 ± 0.06% to 13.12 ± 0.16% for the pigmented and non-pigmented aromatic rice.

The ash values were significantly different amongst the seven fractions. An increasing trend was observed with the decrease of the particle size. The first fraction had the lowest ash content of 0.92 ± 0.02 g/D.W. whereas the highest was found for F7 (4.13 ± 0.20 g/D.W.) (Table S1). Verma and Srivastav [21] reported different ash content for aromatic and non-aromatic Indian rice, varying from 0.35 ± 0.05% in Sarbati rice to 0.73 ± 0.05% in Khushboo rice. These results could be explained by concentration of the compounds in the bran layers of the caryopsis.

The fat concentrations varied between of 5.10 ± 0.16 g/D.W. in F4 and 5.56 ± 0.14 g/D.W. in F5 (Table S1). Chagam et al. [25] reported a lipid content of 3.33 ± 0.20% in the raw Chak-hao Amubi variety. Similar results for the lipid concentration were reported by Saikia et al. [23] for the Poreiton Chakhao pigmented aromatic rice of 2.1 ± 0.08%.

The protein content also increased from 8.69 ± 0.12% in F1 to 10.87 ± 0.15% in F7. Saikia et al. [23] reported for the milled Chakhao amubi rice protein content of 8.8%, whereas Verma and Srivastav [21] suggested different protein content ranging from 6.87 ± 0.10% to 9.51 ± 0.25%. The increase of the protein contents as increasing the sieving is due to the concentration of the protein in the endosperm. Itani et al. [26] reported that most of the protein fractions and fats are mainly located in the rice germ. Therefore, removing the bran and part of the rice endosperm through polishing may cause the decrease of protein concentration [27].

The highest contents in fiber were obtained in F4 and F5 (3.33 ± 0.69 % and 3.56 ± 1.17%, respectively), which are an indicative of fiber’s location in the aleuronic layer of the grain. Murfidin et al. [28] reported a lower fiber content of pigmented rice varieties ranging from 0.66 to 1.14%. 

A decreasing trend for the carbohydrate content from 74.69 ± 0.06 g/D.W. to 65.16 ± 0.08 g/D.W. found in F7 was observed (Table S1). However, it can be considered that all the flour fractions are good sources of carbohydrates. Relative higher contents of carbohydrates were reported by Verma and Srivastav [21] in eight varieties, varying from 78.38 ± 0.12% in Swetganga rice and 82.70 ± 0.24% in Badshah Bhog rice. 

Based on the obtained results, it can be concluded that sieving up to F5, the ash, fat, protein and fiber content increases, while sieving further to the seventh fraction leads to a decrease in the fiber and carbohydrates content. 

### 3.2. Phytochemical Content

The phytochemical content of black rice fractions was previously reported by Bolea et al. [29]. These authors reported that all the seven fractions of black rice contained a significant amount of TPC, ranging from 199.14 ± 0.097 mg GAE/100 g D.W. (in integral flour) to 248.1 ± 3.01 mg GAE/100 g D.W. (in F1) and 483 ± 0.13 mg GAE/100 g D.W. (in F4). Sieving up to F4 led to an increase in TPC being approximately two-times higher than TPC in F4, whereas up to F7 led to an increase of 1.44. Gong et al. [30] reported significant lower TPC value for different brown rice varieties that ranged from 72.45 to 120.13 mg of GAE/100 g. Significantly higher values were reported by Zhang et al. [7], varying between 2365 to 7367 mg of GAE/100 g D.W. among the 12 black rice varieties. 

The highest TFC value was found in the initial flour, of 211.14 ± 0.11 mg CE/100 g D.W., whereas sieving caused a significant decrease ranging from 84.97 ± 4.31 mg CE/100 g D.W. in F1 to 87.9 ± 6.05 mg CE/100 g D.W. in F5 and 76.82 ± 2.91 mg CE/100 g D.W. in F7 [31]. A significant decrease was observed in F2, followed by an increase in the F3–F6. Zang et al. [7] reported higher TFC values, ranging from 3596 ± 2020 mg QE/100 g in Heinuo 9933 variety to 85620 ± 433 mg QE/100 g in Heijing 72 variety. 

Bolea and Vizireanu [31] reported a TAC, in mg C3G mg/100 g D.W. of 9.20 ± 1.50 for the integral flour, whereas the lowest TAC value of 5.80 ± 1.20 was found in F1, followed by F2 with 5.20 ± 1.30, F3 with 3.40 ± 0.10 and F7 with 2.4 ± 0.1, respectively. Significantly higher values ranged from 1231 to 5101 mg of C3G /100 g of D.W. were reported by Zang et al. [7]. 

The antioxidant activity was approximately 71% in whole flour, whereas sieving caused a decrease in antioxidant activity. Therefore, it can be appreciated that grinding and sieving generally lead to a decrease in the biologically active compounds, except for the total polyphenols.

### 3.3. Proteins Patterns of Black Rice

The rice flour with the highest protein concentration was further used to identify the main proteins fractions. It appeared that high amounts of polymers and monomers were concentrated into the stream that had the particle size lower than 90 µm (F7) after the sieving process of black rice. 

**Figure 1:**
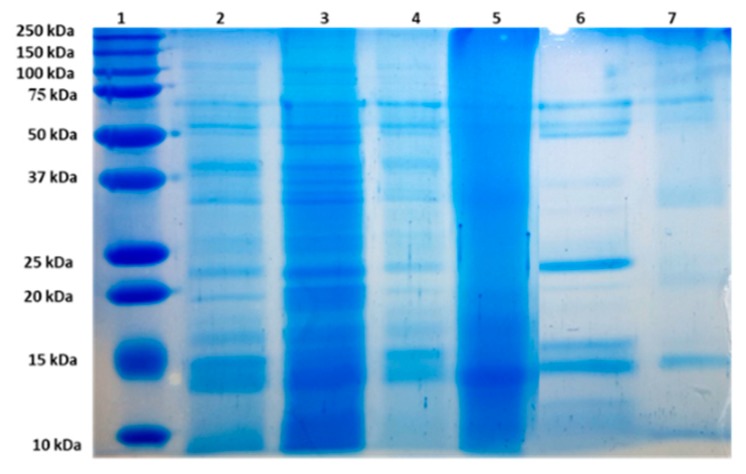
SDS-PAGE (4.5%–15%) profile of the black rice flour proteins (lane 2), of F7 proteins and of albumin, glutelin, globulin and prolamin extracts (lanes 3–7). Lane 1- Precision Plus Protein Dual Extra.

The SDS-PAGE pattern of the proteins from the F7 stream obtained from black rice flour is presented in Figure 1. Based on the solubility differences, the proteins from rice can be classified as ALB which are water soluble, GLU which are alkali soluble, GLO soluble in salt solutions and prolamins soluble in aqueous alcohol. The first vegetal storage proteins classification, provided by Osborn in 1924, depicted the main fractions as albumins, globulins, prolamins, and glutelins. The major fractions of the rice endosperm proteins are GLU (66%–78%), followed by GLO (9.6%–10.8%), ALB (3.8%–8.8%), and prolamin (2.6%–3.3%). When investigating the brown rice which was reported to be rather similar to black rice, Asano et al. [25] showed that most of the proteins are alkali soluble ones (66.0%–67.7%), being followed by ALB and GLO (18.8%–20.8%), and finally the prolamins which are in the lowest concentration (12.5%–14.5%). Zhao et al. [32] suggested that the rice endosperm contained 81.9% GLU and 13.2% GLO, while the rice dreg contained 84.6% of GLU. ALB and prolamin represented minor components for this particular rice. The ALB fractions were 7.7% for the rice dreg, being higher than in the rice endosperm, however, the globulin fraction in the rice dreg was lower than average.

By assessing the results of the SDS-PAGE analysis presented in Figure 1, it can be seen that the molecular weights of ALB fractions were distributed in the range of 13–16 kDa, 20–25 kDa, and 35–50 kDa. GLU had bands around 13–25 kDa and 35 kDa regions, whereas the GLO bands were estimated to correspond to 12–17 kDa, 20–27 kDa, and 50–70 kDa. Finally, the profile obtained for the prolamin extract was characterized by the existence of two intense bands around 10 and 15 kDa (Figure 1).

The molecular weights and the localization of rice proteins were reported by many researchers [2,33]. Generally, 60%–80% of the total seed protein found in rice, is identified as GLU, which are classified based on their amino acid sequence similarity into 4 groups, namely GluA, GluB, GluC, and GluD [34]. Moreover, the GLU subunits were divided into pro-glutelin that have molecular weights of 55 kDa, a large GLU subunit with a molecular weight of 34 kDa and a small subunit of glutelin, with 21 kDa [34]. However, under reducing conditions, the SDS-PAGE analysis revealed only two bands corresponding to proteins of 20–23 kDa and 32–35 kDa, respectively. 

Li et al. [35] used the immunoblotting analysis to quantify the seed storage GLU and prolamins, and reported a molar ratio of 1.7 for the 10 days old seeds. The purified prolamins fraction was distinguished in the SDS-PAGE as a unique band that had the molecular weight of 15 kDa.

On the other hand, the ALB fraction from the seed endosperm had the major molecular band around 20 kDa, while the band identified in the case of GLO corresponded to the molecular weights to 15, 25.5, and 200 kDa [35]. It has been reported that, these types of GLO were also found in the soybean, represented by 7S-globulin (57 and 43 kDa) and 11S globulin (22–23 kDa and 37 kDa) [33].

### 3.4. Kinetics of Phytochemical Thermal Degradation in Different Milled Fractions

The linear regression for the thermal degradation of TPC, TAC, TFC, and DPPH-RSA for the integral flour and F4 were confirmed by [18]. For the other fractions, the phytochemical thermal degradation followed a first-order kinetic model and was described in terms of degradation rate *k* (1/min) and activation energy (*E_a_*). Our results are in good agreement with previous studies that reported the use of the first order kinetic model which described the thermal degradation of phytochemical from black rice [31].

By comparing the thermal degradation rate constants of all the studied compounds (Table 1), it could be appreciated that regardless the fraction, anthocyanins degraded faster. The linear regression for the thermal degradation of TAC are shown in Figure S1. The highest degradation rate was found in F1, as the fraction with the lowest anthocyanins content ranged from 17.43 ± 1.01 × 10^−2^ 1/min at 60 °C to 20.42 ± 2.22 × 10^−2^ 1/min at 100 °C. With regards to the integral flour extract, TAC degraded with the *k* vales varying from 0.92 ± 0.56 × 10^−2^ 1/min at 60°C to 1.22 ± 0.87 × 10^−2^ 1/min at 100 °C, while in F4, the *k* values ranged from 5.52 ± 1.07 × 10^−2^ 1/min at 60 °C to 6.61 ± 0.89 × 10^−2^ 1/min at 100 °C [18].

Figure S2 showed the thermal degradation behavior of TPC in all studied fractions of the black rice flour. The *k* and *E_a_* values are given in Table 2. No significant changes concerning the *k* values were found for F1 in the temperature range of 60 to 90 °C. However, it can be observed that the *k* values significantly decreased by increasing the sieving degree. The lowest degradation rate for TPC was found in fractions F4 as reported by Bolea et al. [18], with *k* values ranging from 0.87 ± 0.18 × 10^−2^ 1/min at 60 °C for F4 to 0.82 ± 0.40 × 10^−2^ 1/min for F7 (Table 1). Increasing the temperature up to 100 °C caused an increase of the *k* values, with the lowest value of 1.01 ± 0.11 × 10 ^−2^ 1/min in F4 [18]. In the case of TPC, the lowest *k* values were obtained for the fraction with the highest content of total polyphenols (F4), while the highest values of degradation constants were obtained for the fraction with the lowest content of total polyphenols (F1). 

Figure S3 showed the thermal degradation behavior of TFC in black rice fractions. The highest degradation rate in the case of TFC was observed for F1. These values were significantly higher than those reported by Bolea et al. [18] for TFC for F4 or for the integral flour (Table 1). However, it can be observed that the *k* values do not depend on the flavonoids content, the highest value being registered for F1, whereas the lowest for F5.

Due to the degradation of biologically active compounds, a significant decrease was recorded in the antioxidant activity, with approximately 58% in F1, 48% in F2, 65% in F3, 29% in F4, 32% in F5, and 33% and 43% in F6 and F7, respectively, after a heating process at 100 °C for 20 min. Figure S4 shows the thermal degradation behavior of DPPH RSA in fractions F1 to F7. The antioxidant activity degraded faster in F1, the estimated *k* values ranging from 1.33 ± 0.11 × 10^−2^ 1/min at 60 °C to 2.18 ± 0.32 × 10^−2^ 1/min at 100 °C, whereas the lowest *k* values were estimated by Bolea et al. [18] in F4, ranging from 0.57 ± 0.24 × 10^−2^ 1/min at 60 °C to 1.21 ± 0.85 × 10^−2^ 1/min at 100 °C. 

To estimate the temperature dependences of the *k* values on temperature, the constants obtained from Equation (1) were fitted to an Arrhenius equation. The activation energy values are given in Table 1. It can be observed, that the *E_a_* values for the TPC thermal degradation increased from 7.05 kJ/mol for F1 to 12.58 kJ/mol for F2, decreased for F3 and F4 up to 3.51 kJ/mol and subsequently increased for F5 to F7 up to 18.77 kJ/mol. It can be stated that the *k* values were less dependent on the temperature in F4, therefore TPCs are thermostable in this fraction and less thermostable in the other.

In the case of TFC, an increase from 1.99 kJ/mol in F1 to 10.78 kJ/mol in F2 can be observed in Table 1. The highest temperature dependence and therefore the lowest thermal stability was found for F5, with an *E_a_* value of 21.93 kJ/mol. Flavonoids were found to be more thermostable in the F3 and less stable in F5.

For TAC, the highest temperature dependence and therefore the lowest thermal stability was found for F7, with an *E_a_* value of 13.79 kJ/mol, while the highest thermostability was found for F1, with the lowest *E_a_* value of 4.31 kJ/mol. 

For the antioxidant activity, the *E_a_* values were found to decrease up to F3 (9.39 kJ/mol) from 16.61 kJ/mol in F1 to 8.27 kJ/mol in F2. The F4 presented the highest value and therefore the lowest thermal stability [18], with an *E_a_* value of 19.93 kJ/mol (Table 1). When comparing to integral flour, it seems that the antioxidant activity is more heat stable, since the lowest *E_a_* value was estimated of 7.45 kJ/mol. 

Overall, it can be appreciated that the thermal stability of the biologically active compounds in the different black rice flour fractions depends on the degree of grinding and sieving, the anthocyanins being the compounds that degrade at the highest rate, significantly affecting the antioxidant activity.

### 3.5. The Effect of Temperature on the Black Rice Protein Fractions

The use of proteins as a natural biopolymer is reasonably increasing in the matter of their application in several fields such as food industry, packaging, and environmental protection. In particular, rice proteins have very interesting properties such as having good nutritional, hypoallergenic, and healthful properties for human consumption [36]. However, although rice proteins have high nutritional value and are hypoallergenic and healthful for human consumption, few studies concerning their structural and conformational properties are reported in literature [36,37,38,39,40]. As a consequence, there is limited data in literature regarding the thermal denaturation of rice proteins fractions from the perspectives of their use as food ingredients, such as in gels, puddings, ice creams, and baby formulas. For example, Ju et al. [33] studied the denaturation and hydrophobic properties of rice flour proteins and concluded that heat denaturation of globulin and glutelin resulted in significant increases in surface hydrophobicity. Rice is routinely subjected to various heat treatments during processing such as steaming, drying, tempering, and roasting. These thermal treatments often lead to substantial denaturation or to the unfolding of the proteins native structure [19]. In our study, we investigated the heat-induced changes of the three rice fractions by fluorescence spectroscopy techniques, which enabled us evaluate in detail the folding–unfolding events of the targeted fractions.

#### 3.5.1. Intrinsic Fluorescence

The fluorescence spectroscopy measurements were performed to observe the folding, unfolding events or the conformational changes that affect the microenvironment of tryptophan (Trp) and tyrosine (Tyr) residues found in the ALB, GLO, and GLU fractions extracted from the black rice flour with particles smaller than 90 µm. The excitation wavelength of 292 nm was used to monitor the changes in the vicinity of Trp residues, 280 nm for both Trp and Tyr, and 274 nm for Tyr. 

Emission spectra obtained after the selective excitation of Trp (Figure S5a) indicated that the maximum fluorescence intensity was registered at 358 nm for ALB fraction, 384 nm for GLO, and 360 nm for GLU. According to Shin et al. [40], the high mobility of amino acids that are exposed to a polar environment, is indicated by the red shifts, while the burial in the non-polar environment generates a blue shift in *λ_max_*. Depending on the environmental properties of the protein, Lakowicz [41] showed that the Trp residues can emit from 308 to 352 nm. It has been reported that Trp residues are buried in a non-polar environment if the maximum fluorescence emission (*λ_max_*) is lower than 330 nm. If the *λ_max_* is higher than 330 nm, the Trp is assigned to a polar environment, which in most cases implies a solvent exposure [42]. The significant higher value obtained for the GLO fractions is an indicative of the higher exposure of Trp residues to a polar microenvironment. 

When heating the ALB fraction, small blue-shifts around 1–2 nm in the *λ_max_* at 50 °C and 60 °C were observed, indicating the partial folding of the polypeptide chains, whereas at higher temperatures, a 2 nm red-shift was found, as an unfolding indicative. For the GLO fraction, heating in the temperature range of 50 to 60 °C caused significant 5 nm blue-shifts in the *λ_max_*, followed by 2 nm red-shifts at higher temperatures. In the case of GLU, no significant heat induced changes were observed in the temperature range of 50–70 °C. Heating at temperatures up to 100 °C caused 2 nm red-shifts in *λ_max_*, indicating the unfolding of polypeptide chains. 

When excited at 280 nm, the *λ_max_* corresponding to the maximum fluorescence intensity for the investigated protein fractions were 355 nm for ALB, 357 nm for GLO and 359 nm for GLU (Figure S5b). These values indicated a higher exposure of Trp and Tyr residues to the solvent in the GLU fractions. The unfolding of polypeptide chains was observed for ALB at temperatures higher than 70 °C. GLO unfolded by heating up to 70 °C and folded at higher temperatures, whereas in the GLU fractions, the Tyr and Trp residues seemed to be more exposed at temperatures higher than 80 °C.

When excited at the wavelength of 274 nm, the protein fractions displayed the fluorescence intensity maximum at 355 nm for ALB, at 356 nm for GLO, while in the case of GLU the maximum fluorescence intensity was recorded at 359 nm (Figure S5c). Heating up to 60 °C caused several folding events and unfolding events at temperatures ranging from 70 to 100 °C for the ALB fractions. For the GLO, up to 60 °C, the unfolding of the polypeptides chains was observed, followed by a folding process up to 100 °C, whereas the GLU unfolds at temperatures higher than 60 °C. The red-shifts suggested that the unfolding of the protein structure strengthened the intermolecular β-sheet hydrogen bonds [43].

Based on the *λ_max_* values, it could be appreciated that in the black rice flour fractions, the Trp and Tyr residues are exposed to the solvent, whereas the heat treatment caused the folding at the lower temperatures range and the unfolding at higher temperatures. Heat-induced denaturation and the unfolding of rice protein structures could release more hydrophilic groups [43] at pH 7.0, possibly resulting in the increase of surface hydrophobicity. Our results are in good agreement with those reported by Zhao et al. [44], who suggested that the heat treatments caused the increase of β-turns at the expense of β-sheets and random coils of rice endosperm proteins. Consequently, the partial unfolding process, which is dominant, revealed diverse protein structural changes that are induced by processing and consistent with the protein surface hydrophobicity increase. Ellepola et al. [19] suggested that rice GLO possesses a relatively high thermal stability (with a denaturation temperature of 70.9 ± 0.04 °C for the milled rice flour, 97.6 ± 0.27 °C for crude rice GLO, and 98.5 ± 0.39 °C for purified GLO), suggesting that the protein could retain its functionality in heat-processed rice products.

#### 3.5.2. Synchronous Fluorescence Spectra

**Figure 2:**
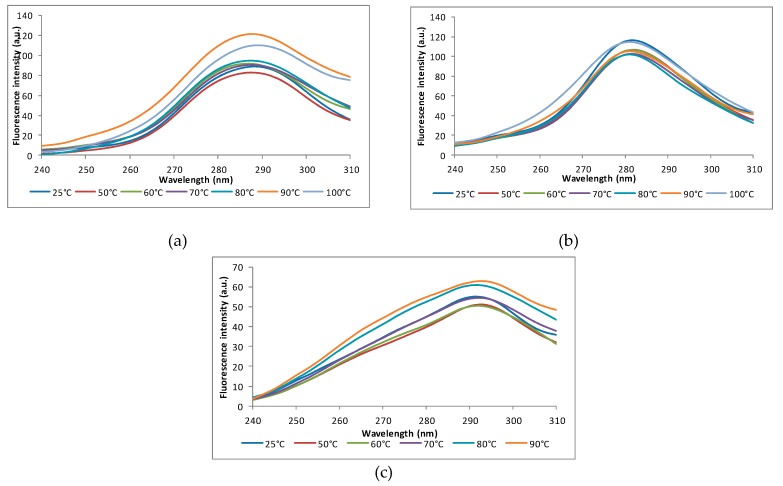
Synchronous fluorescence spectra of the rice proteins at Δλ = 15 nm at different temperatures values: (**a**) albumins, (**b**) globulins, and (**c**) glutenins. Three independent tests were carried out in each case and standard deviation (SD) was lower than 3.5%.

The fluorescence synchronous spectra are another method to investigate the amino acid residues microenvironment by modifying the highest wavelength value (*λ_max_*) obtained at emission, which corresponds to the polarity change around the hydrophobic groups of the molecule [45]. In the tested temperature range, the synchronous spectra at Δλ of 15 nm indicated the presence of a red shift of 3 nm for the ALB fraction (from 286 nm at 25 °C to 289 nm at 100 °C), and small blue shifts of 1 nm and 2 nm for the GLO and GLU fractions (from 282 at 25 °C to 281 nm at 100 °C and from 295 nm to 293 nm, respectively) (Figure 2). The red-shifts for the ALB fractions suggested the exposure of the Tyr residues to a more polar microenvironment, whereas the blue shifts in Δλ for GLO and GLU are an indicative of the heat-induced burial of Tyr residues to a more non-polar microenvironment.)

**Figure 3:**
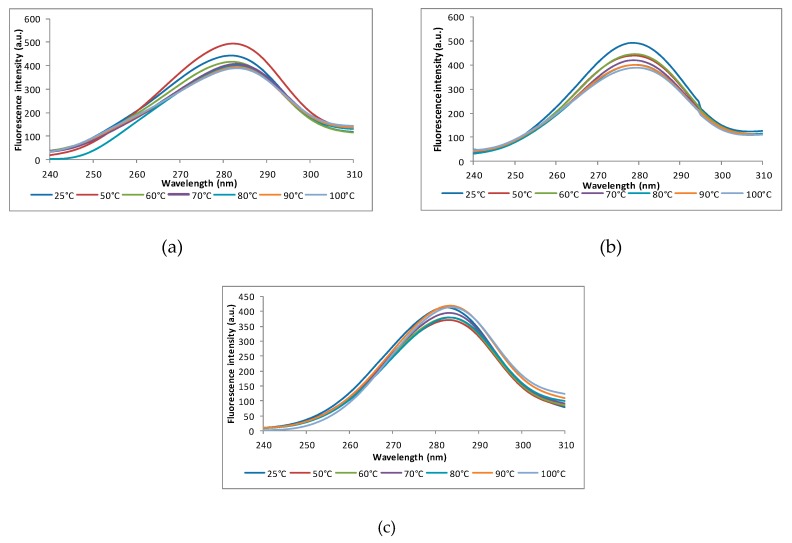
Synchronous fluorescence spectra of the rice proteins at Δλ = 60 nm at different temperatures values: (**a**) albumins, (**b**) globulins, and (**c**) glutenins. Three independent tests were carried out in each case and SD was lower than 3.5%.

Furthermore, the synchronous spectra at Δλ of 60 nm showed small red shifts for all the tested fractions when rising the temperature from 25 °C to 100°C (from 282 nm to 283 nm, from 278 nm to 280 nm, and from 282 nm to 284 nm for ALB, GLO, and GLU, respectively), therefore indicating the exposure of the Trp residues to a more polar microenvironment (Figure 3).

Wherefore, it can be appreciated that the thermal treatment induced the exposure of Tyr and Trp residues in the ALB fractions, whereas for the GLO and GLU fractions, the Tyr residues were buried in a more non-polar microenvironment, while Trp residues became exposed.

#### 3.5.3. Quenching Experiments

In order to check the accessibility of fluorescent residues of black rice proteins fractions to different quenchers, experiments with acrylamide and KI were performed. Acrylamide and KI are external quenchers (charged and non-charged) used to analyze the solvent accessibility and the polarity of the microenvironment close to the Trp residues. The selection of the two quenchers was based on the different accessibility of the quenchers, as acrylamide quenches the exposed and partially exposed Trp residues, while KI quenches only the fluorescence of the exposed Trp located at or near to the surface of the molecules. As expected, when quenching with acrylamide, the *K_SV_* were higher than those obtained with KI, regardless of the applied heating conditions (Table 2), thus indicating that the *K_SV_* value for the terminal Trp residues are higher than those for the buried ones.

From Table 2 and Table 3 it can be observed a significantly higher Trp residues accessibility for acrylamide and KI in GLO, followed by GLU and ALB fractions. Heating caused important conformational changes, as indicated by the sequential decrease of *K_SV_* values at 50 °C for GLO and GLU and an increase for the ALB fractions. The highest values for the acrylamide quenching constants were measured after a thermal treatment at 60°C for ALB (15.11 ± 3.32 mol^−1^ L), at 100 °C for GLO (19.63 ± 2.70 mol^−1^ L) while for the GLU fraction the highest value was recorded at 100 °C (14.15 ± 1.67 mol^−1^ L) (Table 2). In the case of ALB fractions, heating at temperatures higher than 60 °C had no significant effect on the quenching constants values. However, the accessibility of acrylamide to Trp was higher when heating comparing to 25 °C. Therefore, it can be appreciated that the conformational transition of the polypeptide chains decreases the distance between the Trp residues and the quenching agent. The increased *K_SV_* values at high temperatures indicated several structural rearrangements as well as the partial exposure of Trp residues within the protein molecules.

A different thermal behavior was observed for GLO and GLU (Table 2), with a decrease at 50 °C, followed by an increase at 60 °C and 70 °C for GLU. In the temperature range of 70–80 °C, the *K_SV_* values decreased, whereas at higher temperature increased again. For all the rice protein fractions, the *K_SV_* values variations indicated the sequential character of the structural and conformational changes being thermally induced. 

For the KI quenching experiments, the maximum *K_SV_* value for ALB was calculated at 100 °C (6.47 ± 0.53 mol^−1^ L) and the minimum value was recorded at 80 °C (4.70 ± 0.57 mol^−1^ L). For the GLO fraction, the maximum *K_SV_* was found at 70 °C (6.65 ± 1.80 mol^−1^ L) and the minimum at 80 °C (4.53 ± 1.50 mol^−1^ L), while the highest value for the GLU fraction was recorded at 90 °C (4.38 ± 1.15 mol^−1^ L) and the lowest one at 70 °C (1.37± 0.42 mol^−1^ L) (Table 3). It seems that Trp residues are more exposed to the solvent at pH 7.0 and 25 °C in the GLO and less exposed in the GLU fractions. For the ALB and GLU fractions, heating caused an increase in regards to the accessibility of Trp with KI, which could suggest an unfolding of the molecule, with the exception of 70 °C. Within the tested temperature range, local heat-induced conformational changes that bury the Trp residues from the molecule surface occur in the GLO fractions.

## 4. Conclusions

In this study, detailed information on the thermal stability of phytochemical and protein fractions was provided, study that was based on the kinetic studies and fluorescence spectroscopy approaches, from the perspectives of preserving the phytochemical content during the industrial processing. Significant phytochemical content was observed in all the seven black rice milled fractions, hence supporting its potential use as functional ingredients in food and related applications, such as pharmaceutical. The thermal degradation of total polyphenols, anthocyanins, flavonoids, and antioxidant activity for each fraction was described by a first order kinetic model in terms of degradation rate and activation energy. Significantly, the phytochemical thermal stability in the different black rice flour milled fractions depended on the degree of grinding and sieving, the anthocyanins being the most heat labile and their degradation affecting the antioxidant activity.

The SDS-PAGE analysis revealed the presence of the albumin fractions, with molecular weights ranging from 13 to 50 kDa, glutelin between 13–25 kDa and 35 kDa, whereas the globulin bands were estimated to correspond to 12–17 kDa, 20–27 kDa, and 50–70 kDa. 

Thermal denaturation studies were performed to observe the conformational changes that involve the local reorientation of the tryptophan and tyrosine residues. The intrinsic fluorescence experiments revealed that tryptophan and tyrosine residues are exposed to the solvent at 25 °C, whereas the heat treatment caused a folding process at the lower temperatures and an unfolding process at higher temperatures. Quenching studies revealed the highest accessibility of the intrinsic chromophores to acrylamide for the globuline fractions, whereas the variation of the quenching constants values pointed out the sequential character of the structural and conformational changes induced by the thermal treatment. These results may be further used in the food industry and other food or nonfood sectors to formulate new products with high functionality.

## Figures and Tables

**Table 1:** Estimated kinetic parameters (rate constant—*k* and activation energy *E_a_*) of phytochemicals thermal degradation in black rice sieving fractions extracts.

Compounds	Temperature ᵒC	F1	F2	F3	F5	F6	F7
		k∙10^−2^ (1/min)					
TAC	60	17.43 ± 1.01 ^a^	10.89 ± 1.70 ^b^	3.75 ± 1.11 ^a^	4.01 ± 0.91 ^c^	5.61 ± 1.26 ^a^	3.01 ± 1.23 ^b^
	70	18.42 ± 1.15 ^a^	9.51 ± 1.72 ^b^	4.26 ± 0.10 ^a^	13.35 ± 1.53 ^b^	5.12 ± 1.21 ^a^	2.99 ± 0.82 ^ab^
	80	18.51 ± 1.14 ^a^	9.76 ± 2.81 ^b^	4.72 ± 1.21 ^a^	13.28 ± 1.62 ^ab^	5.08 ± 1.42 ^a^	3.38 ± 1.25 ^ab^
	90	20.38 ± 1.28 ^a^	12.45 ± 1.71 ^b^	5.59 ± 0.10 ^a^	13.67 ± 1.89 ^ab^	5.34 ± 1.12 ^a^	3.96 ± 1.23 ^ab^
	100	20.42 ± 1.22 ^a^	17.11 ± 2.20 ^a^	5.55 ± 0.41 ^a^	15.03 ± 1.24 ^a^	5.61 ± 1.10 ^a^	5.15 ± 1.21 ^a^
*E_a_* (kJ/Mol)		4.31 ± 0.98	4.69 ± 0.93	10.77 ± 1.53	10.18 ± 2.89	11.99 ± 1.81	13.79 ± 0.20
TPC	60	2.37 ± 0.24 ^a^	1.88 ± 0.12 ^d^	1.88 ± 0.10 ^c^	0.89 ± 0.31 ^c^	0.92 ± 0.11 ^a^	0.82 ± 0.20 ^c^
	70	2.53 ± 0.25 ^a^	2.23 ± 0.20 ^c^	2.07 ± 0.22 ^bc^	1.15 ± 0.31 ^b^	1.19 ± 0.12 ^a^	0.92 ± 0.11 ^c^
	80	2.97 ± 0.37 ^a^	2.59 ± 0.21 ^c^	2.12 ± 0.92 ^ab^	1.22 ± 0.16 ^b^	1.24 ± 0.31 ^a^	1.31 ± 0.11 ^b^
	90	3.23 ± 0.27 ^a^	2.58 ± 0.63 ^a^	2.11 ± 0.52 ^ab^	1.31 ± 0.23 ^ab^	1.35 ± 0.22 ^a^	1.45 ± 0.10 ^b^
	100	3.23 ± 0.17 ^a^	2.71 ± 0.10 ^b^	2.32 ± 0.20 ^a^	1.58 ± 0.11 ^a^	1.63 ± 0.31 ^a^	1.63 ± 0.11 ^a^
*E_a_* (kJ/Mol)		7.05 ± 1.33	12.58 ± 0.76	4.57 ± 1.68	13.16 ± 1.86	13.18 ± 1.99	18.77 ± 2.35
TFC	60	4.83 ± 0.40 ^b^	3.24 ± 0.30 ^d^	2.67 ± 0.21 ^a^	1.10 ± 0.41 ^d^	2.56 ± 0.11 ^bc^	2.25 ± 0.20 ^a^
	70	4.92 ± 0.41 ^b^	3.38 ± 0.63 ^d^	2.69 ± 0.28 ^a^	1.77 ± 0.51 ^c^	2.54 ± 0.23 ^c^	2.23 ± 0.29 ^a^
	80	5.02 ± 0.04 ^b^	3.77 ± 0.30 ^c^	2.58 ± 0.10 ^b^	1.95 ± 0.10 ^bc^	2.71 ± 0.20 ^ab^	2.18 ± 0.12 ^a^
	90	5.24 ± 0.50 ^b^	4.28 ± 0.40 ^b^	2.71 ± 0.24 ^a^	2.23 ± 0.23 ^b^	2.97 ± 0.21 ^ab^	2.14 ± 0.12 ^a^
	100	5.31 ± 0.50 ^a^	4.88 ± 0.40 ^a^	2.78 ± 0.62 ^a^	2.83 ± 0.52 ^a^	3.01 ± 0.32 ^a^	2.11 ± 0.22 ^a^
*E_a_* (kJ/Mol)		1.99 ± 0.70	10.78 ± 1.25	0.90 ± 1.51	21.93 ± 3.43	3.84 ± 2.21	1.98 ± 0.87
DPPH	60	1.33 ± 0.11 ^a^	1.28 ± 0.21 ^c^	1.30 ± 0.10 ^b^	0.78 ± 0.21 ^a^	0.69 ± 0.43 ^b^	0.94 ± 0.12 ^bc^
	70	1.40 ± 0.10 ^a^	1.51 ± 0.10 ^bc^	1.42 ± 0.31 ^a^	0.73 ± 0.22 ^a^	0.76 ± 0.32 ^b^	0.92 ± 0.63 ^c^
	80	1.49 ± 0.31 ^a^	1.51 ± 0.12 ^bc^	1.48 ± 0.21 ^a^	0.78 ± 0.33 ^a^	0.76 ± 0.31 ^b^	1.01 ± 1.10 ^bc^
	90	2.14 ± 0.20 ^a^	1.65 ± 0.20 ^a^	1.49 ± 0.10 ^a^	0.86 ± 0.2 ^a^	1.01 ± 0.61 ^a^	1.10 ± 1.00 ^b^
	100	2.18 ± 0.32 ^a^	1.84 ± 0.10 ^b^	2.18 ± 0.31 ^a^	1.22 ± 0.52 ^a^	1.26 ± 0.51^a^	1.63 ± 1.10 ^a^
*E_a_* (kJ/Mol)		16.61 ± 1.98	8.27 ± 1.30	9.39 ± 1.87	9.82 ± 1.23	16.61 ± 3.98	13.80 ± 1.49

For the same fraction, means on the same row that do not share the same letter are significantly different at *p* < 0.05 based on the Tukey method.

**Table 2:** The Stern Volmer quenching constant (*K_SV_*) with acrylamide at different temperatures.

Temperature °C	*K_SV_* (10^−3^ L/mol)		
	Albumins	Globulins	Glutenins
25	9.40 ± 0.55 ^b^	18.07 ± 1.79 ^ab^	13.60 ± 0.94 ^a^
50	11.72 ± 0.47 ^ab^	15.57 ± 0.54 ^ab^	8.55 ± 0.68 ^a^
60	15.11 ± 3.32 ^a^	19.41 ± 0.06 ^a^	12.47 ± 0.04 ^a^
70	13.79 ± 1.11 ^ab^	12.51 ± 0.19 ^b^	13.25 ± 0.46 ^a^
80	14.3 0± 1.84 ^ab^	12.22 ± 1.96 ^b^	10.96 ± 0.67 ^a^
90	13.75 ± 2.84 ^ab^	14.93 ± 0.86 ^ab^	13.79 ± 1.77 ^a^
100	13.52 ± 0.80 ^ab^	19.63 ± 2.70 ^a^	14.15 ± 1.67 ^a^

For each fraction, means on the same row that do not share the same letter are significantly different (*p* < 0.05) based on Tukey method

**Table 3:** The Stern Volmer quenching constant (*K_SV_*) with potassium iodide (KI) at different temperatures.

Temperature °C	*K_SV_* (10^−3^ L/mol)		
	Albumins	Globulins	Glutenins
25	4.92 ± 0.87 ^a^	7.03 ± 1.17 ^a^	1.37 ±0.42 ^b^
50	5.04 ± 0.79 ^a^	6.56 ± 0.21 ^a^	2.37 ± 0.11 ^ab^
60	5.65 ± 1.01 ^a^	5.10 ± 0.95 ^a^	1.99 ± 0.51 ^b^
70	5.79 ± 1.41 ^a^	6.65 ± 1.80 ^a^	1.29 ± 0.65 ^b^
80	4.70 ± 0.57 ^a^	4.53 ± 1.50 ^a^	1.81 ± 0.32 ^b^
90	6.41 ± 1.00 ^a^	5.43 ± 2.26 ^a^	4.38 ± 1.15 ^a^
100	6.47 ± 0.53 ^a^	6.21 ± 1.10 ^a^	3.12 ± 0.91 ^ab^

For each fraction, means on the same row that do not share the same letter are significantly different (*p* < 0.05) based on Tukey method.

## Supplementary Materials

The following are available online at https://www.mdpi.com/2304-8158/8/4/131/s1, Figure S1: Isothermal degradation of TPC in black rice fractions F1 (a), F2 (b), F3 (c), F5 (d), F6 (e), F7 (f) extracts treated at different temperatures (♦ 60 °C, ■ 70 °C, ▲ 80 °C, ◇ 90 °C, ☐ 100 °C), Figure S2: Isothermal degradation of TAC in black rice fractions F1 (a), F2 (b), F3 (c), F5 (d), F6 (e), F7 (f) extracts treated at different temperatures (♦ 60 °C, ■ 70 °C, ▲ 80 °C, ◇ 90 °C, ☐ 100 °C), Figure S3: Isothermal degradation of TFC in black rice fractions F1 (a), F2 (b), F3 (c), F5 (d), F6 (e), F7 (f) extracts treated at different temperatures (♦ 60 °C, ■ 70 °C, ▲ 80 °C, ◇ 90 °C, ☐ 100 °C), Figure S4: Isothermal degradation of DPPH in black rice fractions F1 (a), F2 (b), F3 (c), F5 (d), F6 (e), F7 (f) extracts treated at different temperatures (♦ 60 °C, ■ 70 °C, ▲ 80 °C, ◇ 90 °C, ☐ 100 °C), Figure S5: The emissions spectra of the rice proteins at 25 °C. The excitation wavelength was 274 nm (a), 280 nm (b), and 292 nm (c) and the spectrums were collected between 310 and 420 nm. Three independent tests were carried out in each case and SD was lower than 10%, Table S1: Proximate compositions of the black rice flour fractions.
