# Supplementary material for: Process-Structure-Function in Association with the Main Bioactive of Black Rice Flour Sieving Fractions
Source: Foods. 2019 Apr 18;8(4):131. doi: 10.3390/foods8040131 (PMC6518182; doi:10.3390/foods8040131)
Supplement: Supplementary file 1 [file foods-08-00131-s001.pdf]

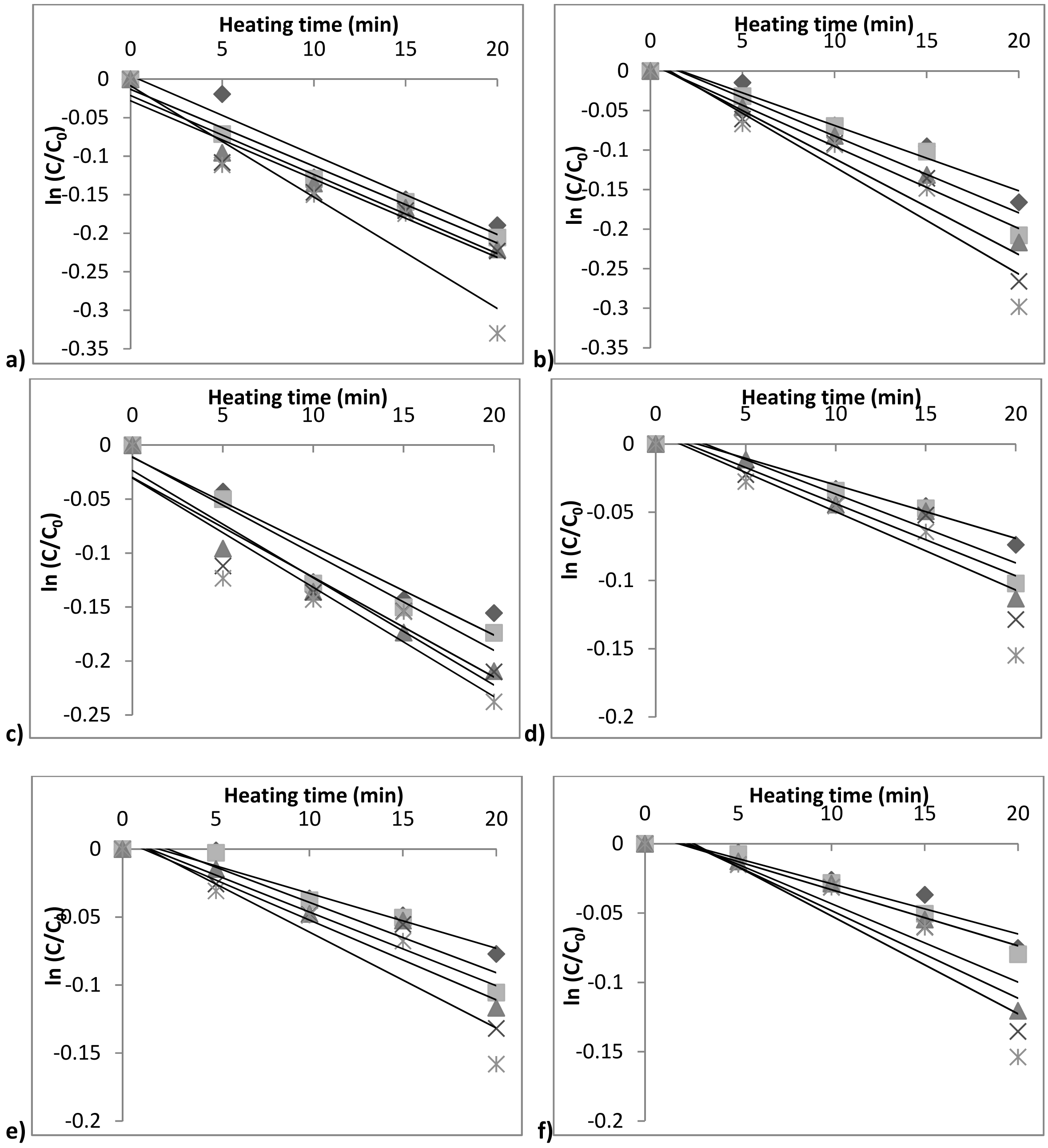

FIGURE S1. Isothermal degradation of TPC in black rice fractions F1 (a), F2 (b), F3 (c), F5 (d), F6 (e), F7 (f) extracts treated at different temperatures (♦ 60 ºC, ■ 70 ºC, ▲ 80 ºC, ◇ 90 ºC, ☐ 100ºC).

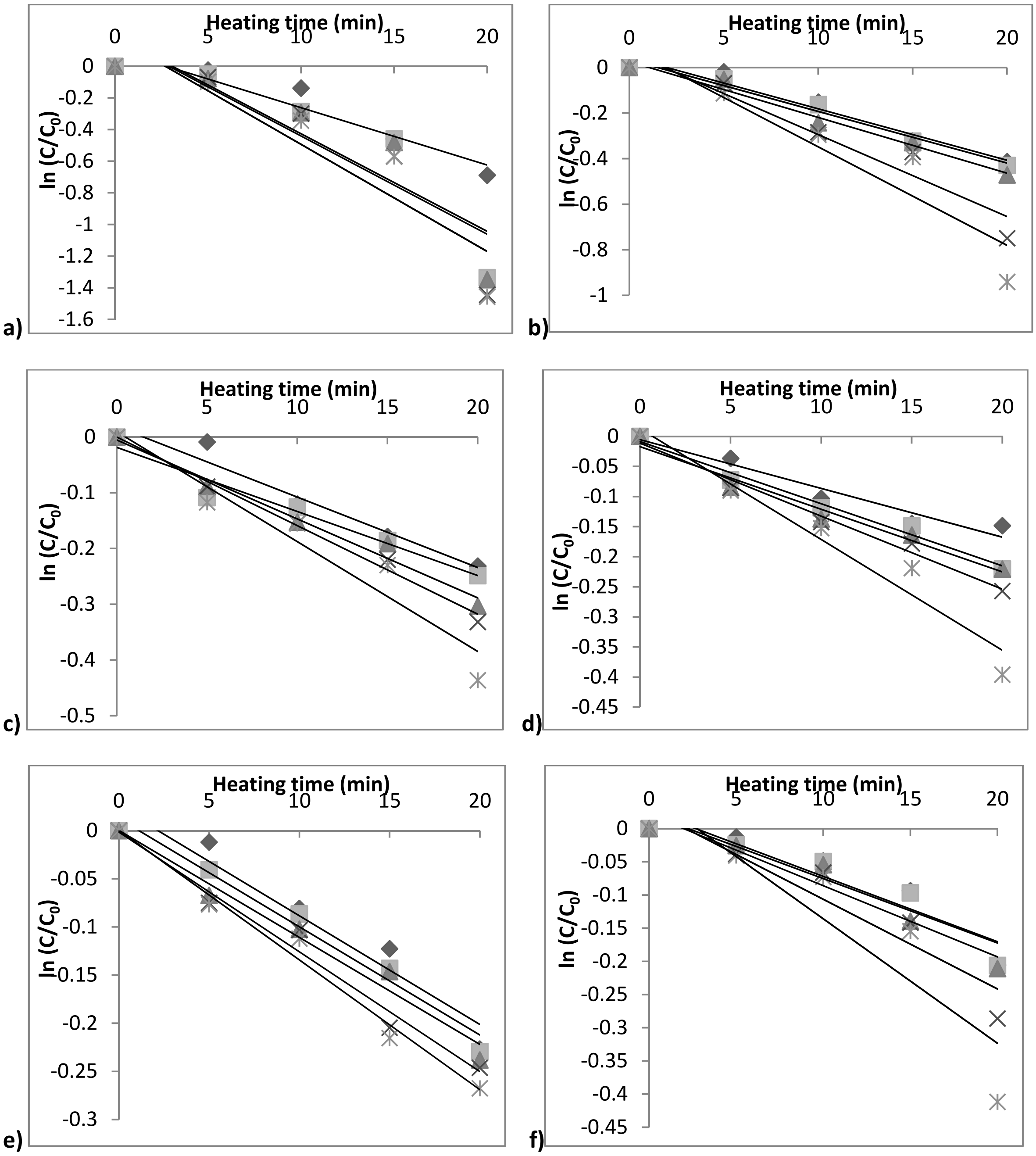

FIGURE S2. Isothermal degradation of TAC in black rice fractions F1 (a), F2 (b), F3 (c), F5 (d), F6 (e), F7 (f) extracts treated at different temperatures (♦ 60 ºC, ■ 70 ºC, ▲ 80 ºC, ◇ 90 ºC, ☐ 100ºC).

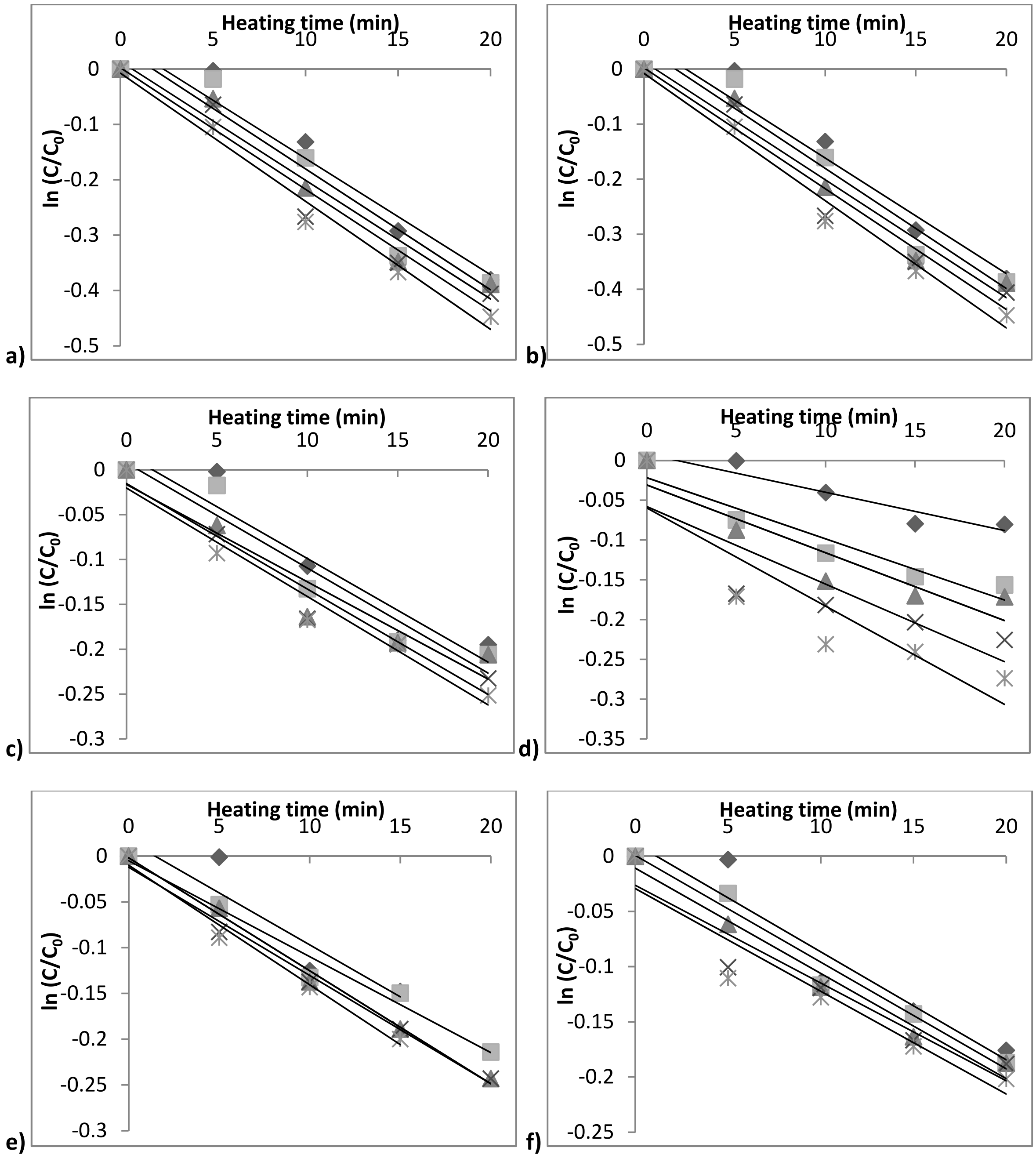

FIGURE S3. Isothermal degradation of TFC in black rice fractions F1 (a), F2 (b), F3 (c), F5 (d), F6 (e), F7 (f) extracts treated at different temperatures (♦ 60 ºC, ■ 70 ºC, ▲ 80 ºC, ◇ 90 ºC, ☐ 100ºC).

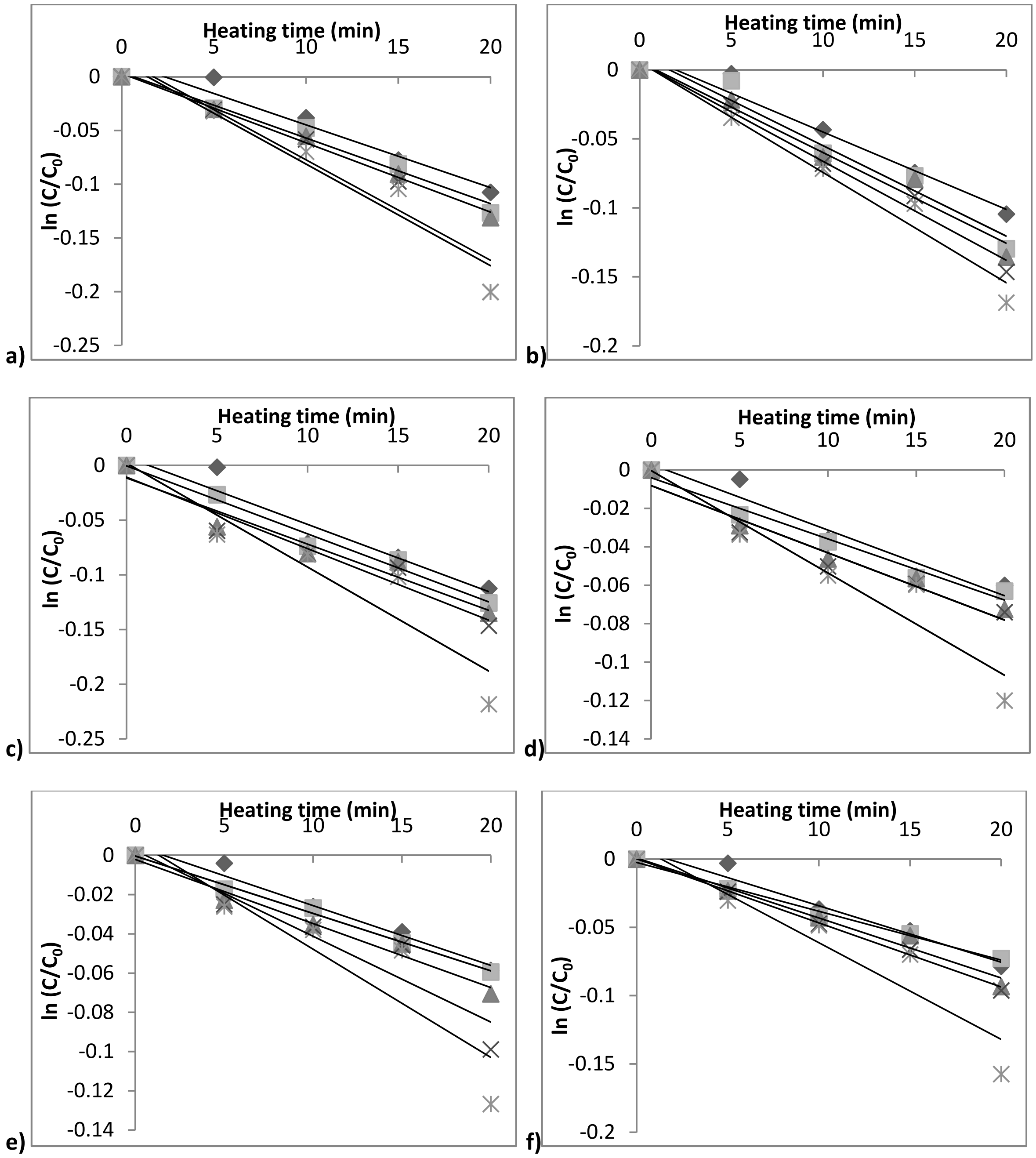

FIGURE S4. Isothermal degradation of DPPH in black rice fractions F1 (a), F2 (b), F3 (c), F5 (d), F6 (e), F7 (f) extracts treated at different temperatures (♦ 60 ºC, ■ 70 ºC, ▲ 80 ºC, ◇ 90 ºC, ☐ 100ºC).

a)

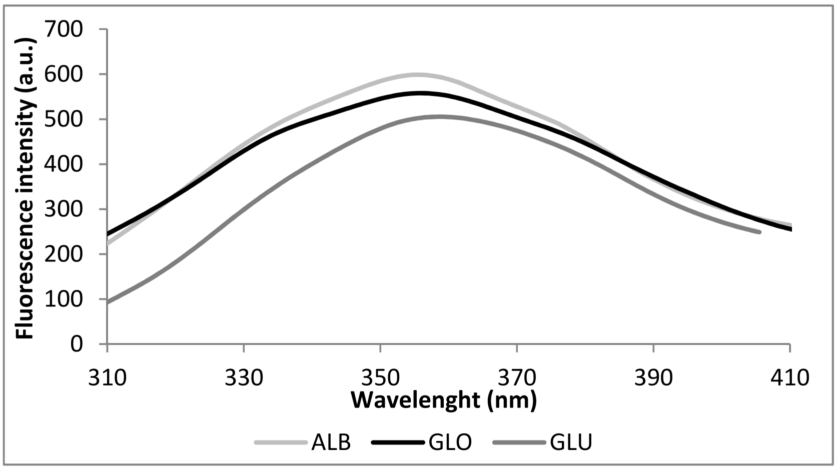

b)

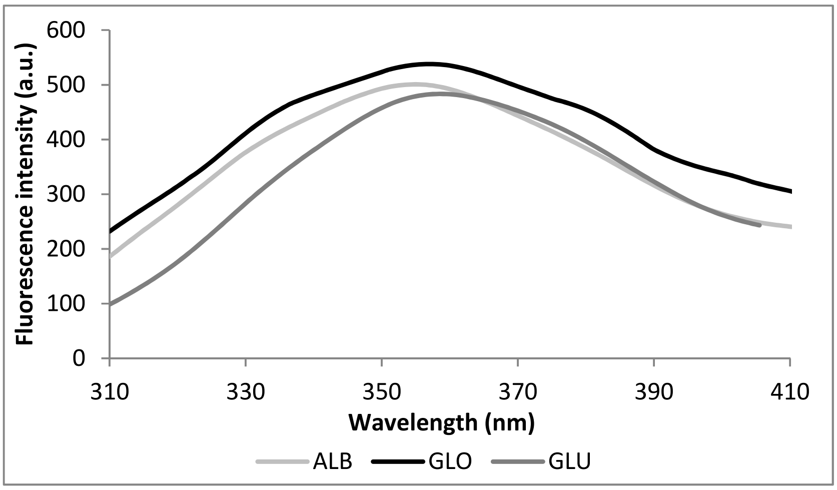

c)

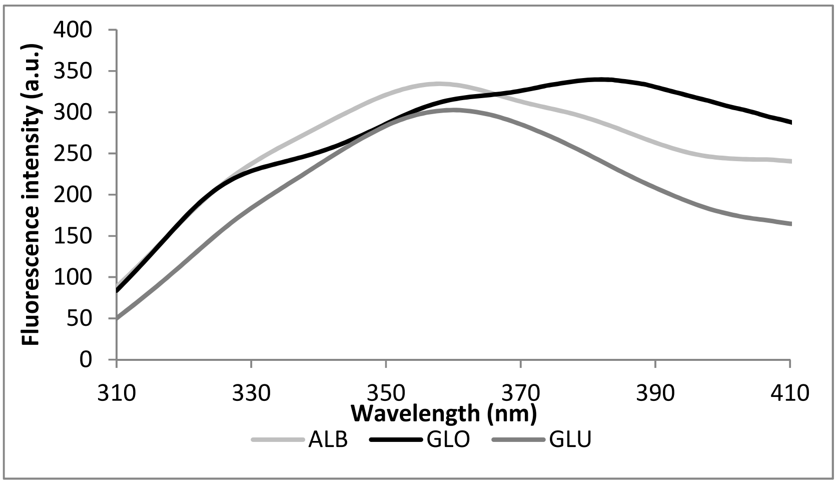

FIGURE S5. The emissions spectra of the rice proteins at 25°C. The excitation wavelength was 274 nm (a), 280 nm (b) and 292 nm (c) and the spectrums were collected between 310 and 420 nm. Three independent tests were carried out in each case and SD was lower than 10%.

TABLE S1. Proximate compositions of the black rice flour fractions

Sample	Moisture %	Ash g/D.W.	Lipid g/D.W.	Protein g/D.W.	Crude fibre %	Carbohydrate g/D.W.
**F1**	11.32±0.04[a]	0.92±0.02	1.32±0.15	8.69±0.12	1.37±0.38	74.69±0.14
**F2**	11.47±0.03	1.69±0.02	1.39±0.23	8.72±0.14	1.97±0.43	72.85±0.11
**F3**	11.27±0.03	2.09±0.06	3.92±0.19	9.20±0.09	2.68±0.4	69.74±0.10
**F4**	11.35±0.06	3.02±0.06	5.10±0.17	9.54±0.05	3.33±0.69	66.01±0.14
**F5**	11.30±0.01	3.37±0.13	5.56±0.14	9.57±0.2	3.56±1.17	65.41±0.09
**F6**	11.22±0.07	3.84±0.16	4.26±0.18	10.52±0.06	1.66±0.44	65.4±0.11
**F7**	11.08±0.03	4.13±0.20	3.91±0.13	10.87±0.15	1.10±0.48	65.16±0.08

[a] Standard error
